# Artificial Oocyte: Development and Potential Application

**DOI:** 10.3390/cells11071135

**Published:** 2022-03-28

**Authors:** Reza K. Oqani, Seongjun So, Yeonmi Lee, Jung Jae Ko, Eunju Kang

**Affiliations:** 1 Department of Biomedical Science, College of Life Science, CHA University, Seongnam 13488, Korea; rkoqani@yahoo.com (R.K.O.); soseongjun7@gmail.com (S.S.); yeonmilee82@chamc.co.kr (Y.L.); 2 Center for Embryo & Stem Cell Research, CHA Advanced Research Institute, CHA Bundang Medical Center, CHA University, Seongnam 13488, Korea

**Keywords:** assisted reproductive technologies, artificial oocyte, oogenesis, stem cells, haploidization of somatic chromosomes

## Abstract

Millions of people around the world suffer from infertility, with the number of infertile couples and individuals increasing every year. Assisted reproductive technologies (ART) have been widely developed in recent years; however, some patients are unable to benefit from these technologies due to their lack of functional germ cells. Therefore, the development of alternative methods seems necessary. One of these methods is to create artificial oocytes. Oocytes can be generated in vitro from the ovary, fetal gonad, germline stem cells (GSCs), ovarian stem cells, or pluripotent stem cells (PSCs). This approach has raised new hopes in both basic research and medical applications. In this article, we looked at the principle of oocyte development, the landmark studies that enhanced our understanding of the cellular and molecular mechanisms that govern oogenesis in vivo, as well as the mechanisms underlying in vitro generation of functional oocytes from different sources of mouse and human stem cells. In addition, we introduced next-generation ART using somatic cells with artificial oocytes. Finally, we provided an overview of the reproductive application of in vitro oogenesis and its use in human fertility.

## 1. Introduction

Estimates show that ~15% of couples at reproductive age suffer from infertility, with the number of infertile couples increasing each year [1]. Infertility is a disease in which a person is unable to have a successful pregnancy after 12 months or more of regular and unprotected sexual intercourse with his/her partner [2]. Although both sexes are affected by infertility, studies have shown that infertility prevalence is relatively higher among women than men [1,3]. Infertility can have a significant and negative impact on families—as well as the social relationships of individuals—and consequently, the development of fertility assistance methods is necessary. In recent years, methods such as ovarian stimulation, in vitro egg maturation, in vitro fertilization, embryo culture, embryo transfer, and intracytoplasmic sperm injection have been widely developed and used to treat infertility. However, only ~70% of infertile couples are able to use this technology, while the remainder suffer from a lack of functional germ cells, meaning alternative solutions are required [4].

A lack of functional germ cells can occur in men as non-obstructive azoospermia and in women as premature ovarian insufficiency (POI) [5]. Autocrine and paracrine factors play an important role in the process of mammalian folliculogenesis, and their dysfunction can lead to the occurrence of POI phenotypes [6]. In addition, more than 100 genes have been identified in which mutations lead to POIs [7]. Genes such as *Nr5a1* [8] and *Nanos3* [9,10] are among them. Mutations in oocyte-specific transcription factor genes such as *Nobox* [11,12] and *Figla* [13], as well as mutations in the *Bmp15* gene, which can severely reduce *Gdf9* expression [14], can cause POI. The only treatment available currently for these patients is through the donation of gametes. However, this method does not lead to the birth of genetically related infants to parents, which is a natural and important concern among many patients.

One promising method that can potentially replace gamete donation is through the use of artificial gametes. These are gametes made through laboratory manipulation of the progenitor cells, pluripotent stem cells or somatic cells, ideally fertilizing and forming an embryo, leading to the birth of a healthy baby. Patients with a history of undergoing anticancer therapies—such as chemotherapy or radiation therapy—and who have lost their gametes before saving them by cryopreservation are advised to use artificial gametes. A healthy same-sex couple, single parents, and postmenopausal women can also benefit from this method.

In addition to serving as a treatment option, artificial gametes will also serve as the cornerstone for basic research. Many studies related to human germ cells cannot be performed on humans because—in addition to ethical considerations—of the unavailability or very limited access to human specimens. The results of animal studies also cannot be directly translated into studies on a human because the process of gametogenesis is not the same across different species. For example, transcriptional regulation in the early stages of primordial germ cell (PGC) development in humans is different from that in mice [15]. However, oocytes and sperm are both derived from stem cells as well as a variety of other sources. In this article, artificial oocytes are focused on demonstrating what sources they can be obtained from.

## 2. Principles of Oocyte Development

Germ cells, which are responsible for creating the next generation, turn into gametes at the end of their differentiation line; together, gametes form the embryo. Germ cells were first observed in mouse embryos on an “embryonic day” (E), specifically on E7–E7.5, as a small cluster of alkaline phosphatase–positive (AP+) cells [16].

**Figure 1:**
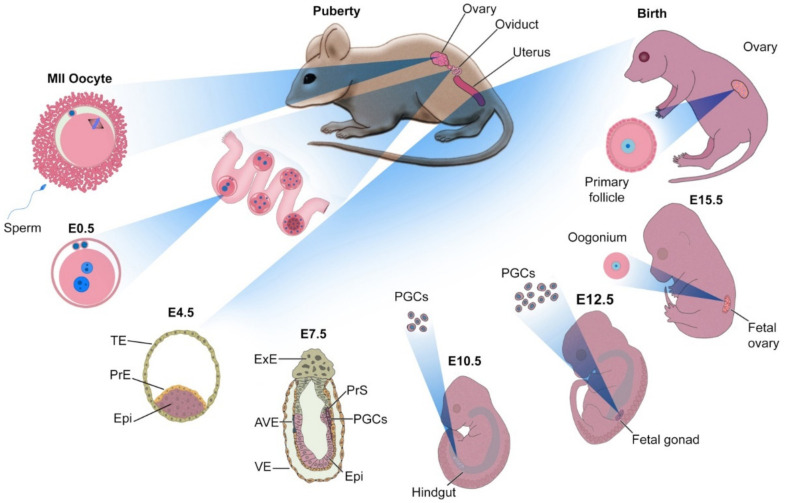
The development of mouse embryo in vivo. Fertilization, cleavage, and morula compaction take place in the oviduct. On an embryonic day 4.5 (E4.5), the advanced blastocyst is positioned in the uterus and is composed of epiblast (Epi), primitive endoderm (PrE), and trophectoderm (TE). On E7.5, the embryo is composed of extra-embryonic ectoderm (ExE), anterior visceral endoderm, visceral endoderm (VE), Epi, and primitive streak (PrS). At this stage, it has begun gastrulation and is connected to the uterus through the ExE. Several cells in the PrS receive signals from ExE and VE that commit them to differentiate into primordial germ cells (PGCs). With the formation of the gut, PGCs migrate through the hindgut, and on the E12.5, they are colonized and multiply in the gonad (which arises from the genital ridge). With the development of the ovary from the gonad, PGCs go through the early stages of differentiation into the oocyte, stopping shortly after birth in the germinal vesicle (GV) stage. With puberty and secretion of gonadotropins, the development of the oocyte is complete and an MII oocyte is formed. This MII oocyte exits the ovary so that it may enter the oviduct, ready for fertilization.

Primordial germ cells (PGCs), which are the precursors to germ cells, originate from epiblast (Epi) cells in the E4.5 blastocyst. During early gastrulation (~E7.5), prior to the formation of the primary germ layers (ectoderm, mesoderm, and endoderm), ~40 AP+ cells accumulate at the base of the incipient allantois in the extra-embryonic mesoderm (ExM) [17]. Figure 1 summarizes the development of PGCs and the generation of oocytes in female embryos.

A distinguishing feature between a group of Epi cells and PGCs are signals that reach the Epi from cells adjacent to the epiblast, which are located in the extra-embryonic ectoderm (ExE). The most important of these signals are the Bone Morphogenetic Protein (Bmp) coding genes, specifically Bmp4, Bmp8b, and Bmp2. Bmp2 is produced by the visceral endoderm (VE); its cooperation with Bmp4—which is produced by the ExE—is essential for the production of PGCs in mice [6]. The Bmp8B, also produced by the ExE, is essential for the production of PGCs [7,8]. These cells then move from the posterior primitive streak to the embryonic endoderm, becoming mobile cells, migrating en masse through the hindgut to the structure that will later develop into the genital ridge, accumulating ~E10.5 in the gonads. At this stage, PGCs undergo morphological changes and stop moving, in turn beginning their sex-specific development into gonocytes.

On E12.5, the sex of the gonocytes is determined. In male embryos, on E10.5, with the expression of the sex-determining region Y (SRY) gene located on the Y chromosome, the expression of the SOX9 gene is stimulated in gonadal somatic cells, resulting in these cells differentiating into Sertoli cells. In the absence of the Y chromosome in the female embryo, between E12.5 and E13.5, PGCs commit to becoming oocytes, which coincides with their transition from mitosis to meiosis. As the embryo continues to develop, the oocytes reach the prophase of meiosis I and stop at this stage, which occurs at roughly the same time as birth. During this period, the oocytes grow dramatically in size, surrounded by layers of somatic cells called granulosa, and a structure called zona pellucida forms between them and the granulosa cells. Oocytes arrest at the prophase of meiosis I. Maintaining an arrested state requires precise control by intra- and extracellular signals.

The most important of these signals are cyclic adenosine monophosphate (cAMP) and cyclic guanosine monophosphate (cGMP). cAMP is built inside the oocyte and its high level is necessary to prevent the progression of meiosis [18]. cGMP is synthesized in granulosa cells and transferred to oocytes. The level of this molecule must also be kept high so that the oocyte remains arrested [19,20]. During puberty, with the secretion of luteinizing hormone (LH), cGMP synthesis in granulosa cells decreases, and cAMP is degraded. Decreased cAMP levels lead to maturation-promoting factor (MPF) activation resulting in resumption of meiosis. This process is discussed in detail elsewhere [21]. During puberty and the secretion of sex hormones, nuclear maturation of the oocyte is complete, transitioning from prophase of meiosis I to meiosis II, and stops in metaphase, at which point the oocyte leaves the ovary and is ready for fertilization.

Knocking out the *Bmp4* alleles results in the complete absence of PGCs in mouse embryos [22]. The allantois is also not formed in these embryos [23,24]. Genomic deletion of downstream factors also leads to impaired germ cell formation. Gene targeting of the Smad protein-coding genes, *Smad1* [25], *Smad4* [26], and *Smad5* [27]—which are downstream effectors of Bmp pathways—also leads to a sharp decrease in the number of PGCs in mice. Bmp4 activates downstream transcription factors by activating the Wnt pathway. One of these factors is called BRACHYURY or T—located downstream of Wnt3 [28]—which activates basic factors for the specification of PGCs, namely Blimp1 [29], Prdm14 [30], and Tfap2c [31], with help from Bmp4. Transcription factor Otx2 also decreases expression in response to Bmp4 in the posterior Epis, which is essential for PGC specification [32].

**Figure 2:**
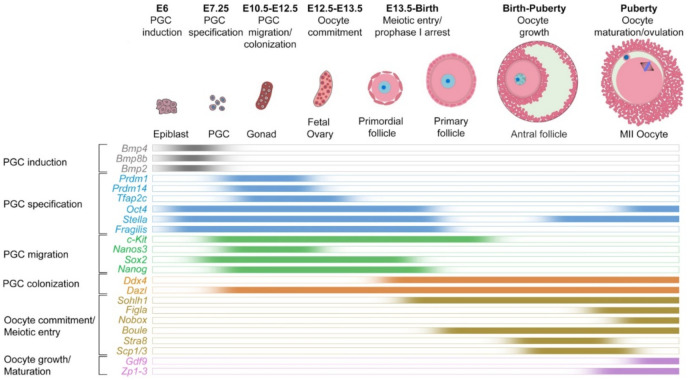
Expression dynamics of key genes regulating the specification of primordial germ cells and the development of oocytes in vivo in mice.

Numerous genes control the transition of PGCs to further stages of differentiation and development. For example, *Oct4* is essential for the specification of PGCs [33]. The expression of genes such as *Stella*, and *Fragilis* increases dramatically at this stage [24]. Further, the migration of PGCs through the hindgut depends on the expression of genes such as c-*Kit* [34] and *Nanos3* [35]. From E13 onward, the expression of *Ddx4* leads to the colonization of PGCs in the gonads and their differentiation to gonocytes [36]. In the gonads and from 13.5 onwards, with the increase in the expression of genes such as *Figla* [37], *Nobox* [11], and *Stra8* [38], gonocytes are sexually identified and become precursors of oocytes, called oogonia. The expression of *Scp* genes indicates meiosis in these oocytes [39]. Finally, the expression of *Gdf9* [40] and *Zp* [41] genes causes the growth and maturation of oocytes and prepares them for ovulation and fertilization. In addition, in the process of oocyte differentiation, growth, and maturation, chromatin undergoes extensive epigenetic changes. These changes are necessary to regulate the expression of genes involved in the growth and maturation of oocytes. He and colleagues have comprehensively reviewed these changes and their consequences in mammalian oogenesis [21]. Figure 2 summarizes the expression dynamics of 23 genes critical for the development of oocytes from PGCs.

## 3. Sources of Oocytes

### 3.1. Maturation of Ovarian Follicles

#### 3.1.1. Ovarian Follicle from Mouse

**Figure 3:**
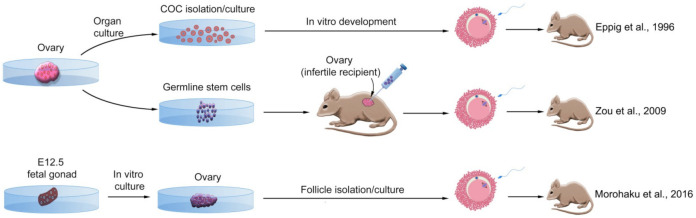
Successful methods of deriving oocytes from mouse ovarian stem cells. Adult ovaries can be a source of cumulus-oocyte complex (COC) after organ culture, or female germline stem cells (GSCs). The GSCs can differentiate into MII oocytes after transplantation into a sterile recipient’s ovary. Fetal gonads can also develop into ovaries in vitro, and secondary follicles can be isolated and cultured from them, allowing them to develop into MII oocytes. All of these strategies led to producing live offspring [43,44,45].

Preliminary experiments on mouse preantral oocytes showed that these cells could grow and develop in vitro, and could produce live pups after maturation and fertilization [42]. These cells were isolated from the ovaries of 12-day-old mice and cultured as oocyte and granulosa cell complexes for 10 days. After IVF and embryo transfer, some of the cells developed to live neonates. The first experiments on follicles were also performed in mice to achieve the complete development of oocytes from the ovaries of neonatal mice [43]. First, the ovaries were cultured for eight days. Then, oocyte-granulosa cell complexes were isolated from the cultured ovaries and re-cultured for further 14 days. A 14-fold increase in PF size was observed after 8 days of culture. More than 90% of the complexes survived and grew in the EGF-containing medium. The medium also supported germinal vesicle breakdown (GVBD) in 30% of oocytes, and 22% of them showed progression to meiosis II by extruding the first polar body. Approximately 42% of these oocytes were fertilized by IVF, forming 2-cell embryos; less than 2% of them reached the blastocyst stage. In addition, out of 190 2-cell embryos transferred to pseudo-pregnant mice, two mice were born to two foster mice, one of which died, while the other survived to reach puberty and fertility. These experiments revealed that complete in vitro development of functional oocytes from PF is possible (Figure 3).

Using αMEM culture medium instead of Waymouth’s—which has a much lower glucose content (5.5 mM instead of 27.5 mM)—and removing the follicle-stimulating hormone (FSH) from the oocyte-granulosa cell complex (OGC) medium, O’Brien et al. significantly increased the amount of GVBD as well as the number of MII oocytes [46]. As a result, the number of 2-cell and blastocyst embryos also increased significantly. This research also showed that depending on the diameter of the follicles, intrinsic culture periods are necessary at the beginning of culture. For example, in the case of dormant primary follicles—which are very small—several stages of organ culture are required before follicles or OGCs begin to grow. These include the majority of intra-ovarian follicles, especially in the cortex.

In a 2-step system, Jin et al. cultured all ovaries of 8-day-old mice for 4 days in an αMEM medium with FSH and insulin. They then isolated the secondary follicles—along with the granulosa cell layer—from the ovaries cultured with alginate beads for 12 days in an αMEM medium and FCS [47]. The cumulus-oocyte complexes (COCs) were then removed from the antral follicles and matured in αMEM plus FCS, hCG, and EGF for 18 h, after which they were fertilized by capacitated sperm in vitro and reached the 2-cell stage. During the 4-day culture of ovaries, the number of primordial follicles—which comprised the 84% majority of the follicles—gradually decreased, becoming secondary follicles.

Mochida et al. introduced a 2-step system for in vitro growth (IVG) of primary/early secondary follicles containing small oocytes, ~45 μm in diameter, with 1–3 layers of granulosa cells that did not require culturing of all or part of the ovaries. This resulted in oocytes in mature follicles completing meiosis as well as fertilizing at a high rate [48]. Early and growing follicles with a diameter of ~60–95 μm were isolated from the ovaries of 6-day-old mice and spent the first stage of culture on a collagen gel. After nine days of culture, the growing follicles were transferred to collagen-coated membranes and allowed to begin the second growth stage. On day 17, the OGC complexes underwent in vitro maturation (IVM). Approximately 90% of oocytes within living follicles showed meiosis II. Through this method, for the first time, living and fertile neonates were obtained from IVG, IVM, and IVF of small follicles.

#### 3.1.2. Ovarian Follicle from Human

Although significant advances have been made in the culture and reconstruction of preantral and antral follicles in different species, the maturation of human follicles has been much more difficult because of the large size and length of time required for development [49]. In 1965, Nobel Laureate Robert Edwards—who later made the first human test-tube baby possible—showed that human antral follicle oocytes could be isolated from ovarian tissue and then matured in a culture medium [50]. Later, Cha et al. showed that oocytes aspirated from antral follicles of infertile women with polycystic ovarian syndrome (PCOS) could mature in a culture medium and produce healthy infants after IVF and embryo transfer [51]. Oocytes aspirated from small antral follicles (~2–10 mm) also produced healthy neonates from women with PCOS after culture, development, IVF, and ET [52,53].

Cultures of biopsied tissues from the ovarian cortex of women aged 25–42 years—immediately after harvest or after 1–4 months of freezing—showed that these tissues, even after 21 days of culture, contained non-atretic primordial, primary, and secondary follicles [54]. Additionally, these follicles could develop into oocytes up to 80 μm in diameter after 2 weeks of culture [55]. These findings showed that culturing small human follicles for developing into oocytes was possible in the laboratory. Later, using a 2-step system similar to that previously performed in mice, Telfer et al. developed preantral follicles from ovarian cortex biopsies in women aged 26–40 years in an ActA-containing medium to oocyte maturation [56]. Following Telfer et al., other groups using a bio-engineered 3D culture system showed that human early secondary follicles had the capability of communicating with somatic cells in vitro and growing to GV oocyte [57]. Further, using a two-step culture, follicles could even grow and produce meiotically-competent MII oocytes [58].

The next finding, namely a three-step culture system developed by McLaughlin et al., would go on to support the development of human PF to MII oocytes [59]. The development of humans follicles in vitro has always been challenging. Follicles need special conditions to develop that meet all their physiological needs, such as hormones, growth factors, and other factors. These environments should support the synthesis of thousands of proteins and RNAs that are essential for fertilization and embryonic development. Accompanying somatic cells is also essential in this process. At present, follicle culture systems are multi-stage and the follicles spend a long time in the culture medium. This can have adverse epigenetic consequences for oocytes. Extensive research is needed to improve the quality of culture systems. Imitation of three-dimensional ovarian conditions, for example, is a solution. For instance, polymers such as alginate hydrogels can mechanically reconstruct the extracellular matrix of the ovary in mice [60,61] and humans [62]. However, since such artificial matrices may not meet all the biological needs of the follicles, in the future the use of biological matrices such as decellularized ovaries may be a better solution for culturing higher quality follicles and obtaining competent oocytes [61].

### 3.2. Ovarian Stem Cells

#### 3.2.1. Ovarian Stem Cells from Mouse

It has been previously assumed that the ability of the ovaries to generate new oocytes is lost at birth or shortly thereafter. It has also been thought that, over time, the number of oocytes decreases due to apoptosis, with the ovaries eventually becoming depleted of germ cells [63,64]. This assumption was taken into question by Johnson et al., who showed that the ovaries of young and adult mice contained mitotically active germ cells that were needed to replace lost follicles [65]. These cells expressed the meiotic entry marker, SYCP3, which decreased in expression with age (Table 1). The results of this study were soon criticized by different research groups; however, the results of subsequent studies partially confirmed it [66]. These cells were also detected in both human [67] and rat [68] ovaries; although, their involvement in oogenesis and functional oocyte production in the ovary continues to be debated. Some researchers still believe that oocytes did not evolve from migratory germline stem cells (GSCs) [69,70,71].

A breakthrough in the field was discovered by Zou et al. [44]. In their study, cells expressing germ cell-specific genes were isolated from immature and mature ovaries and cultured in vitro (Table 1). Cells were first isolated from ovaries of either 5-day-old or adult mice expressing the Mvh protein (also called Ddx4) by immunomagnetic separation. Following this, the proliferation potential was evaluated using BrdU labeling. The labeling test revealed the presence of BrdU-Mvh double-positive cells in the ovarian surface epithelium (formerly known as the germinal epithelium), which further indicated the potential presence of female germline stem cells (FGSCs) in the ovaries. In these cells, the expression of *Oct4*, *Blimp1*, *Dazl*, *Stella*, *Mvh*, *Fragilis*, and *Rex1* genes was detectable, but instead, no expression of *c-kit*, *Figla*, *Sox2*, *Nanog*, *Scp1-3*, or *Zp3* genes was observed. The cells were able to replace lost oocytes after transplantation into the ovaries destroyed with chemotherapy drugs. These new oocytes were able to both fertilize and produce live pups expressing the genetic marker GFP, which was inserted before cell transplantation. Therefore, it is possible to regenerate oocytes in sterile female mice through grafting FGSCs (Figure 3).

In a study by Morohaku et al. (2016), all three events required for a successful in vitro oogenesis were reconstructed namely: the onset of the meiotic phase, follicular assembly, and oocyte growth/maturation [45]. The majority of oocytes produced in this study developed into MII, with ~half of them being fertilized through IVF. The resulting zygotes reached the two-cell stage with a high majority and developed into healthy offspring after transferring to recipients. Moreover, follicles obtained from cryopreserved fetal gonads yielded healthy offspring after development under the same conditions (Figure 3).

A genetic study revealed a direct relationship between oocyte production in adulthood and ovarian activity [73]. Targeted and reversible depletion of pre-meiotic germ cells differentiating into oocytes in HSVtk-transgenic mice reduced the number of oocytes in response to the impaired new oocyte input. However, after ending the depletion protocol, germ cell differentiation resumed, resulting in complete regeneration of the oocyte storage.

In addition, induction of a fluorescent report by activation of Cre-recombinase in Stra8-expressing cells—i.e., new oocytes—showed that induction of this system in adulthood results in the formation of a mosaic store of both old and new oocytes (unlabeled and labeled). Labeled oocytes matured and fertilized, producing offspring that reached adulthood, passing on the reporter to the next generation. These and subsequent studies have shown that GSCs are present in the ovaries of adult mammals and play a direct role in the formation of new oocytes [101].

#### 3.2.2. Ovarian Stem Cells from Human

Based on previous studies, some observations suggest the possible presence of stem cells in the adult human ovarian surface epithelium (OSE) layer. For example, telomerase activity, which maintains telomere length in germline tissues and stem cells, has been detected in OSE, embryos, infants, and adults [102]. High expression of the c-KIT receptor and KIT-ligand/SCF genes has been reported in normal as well as cultured human and bovine OSE [103]. In the OSE cell cultures from the ovaries of postmenopausal women, the differentiation of these cells into oocyte-like cells (OLCs) was reported, indicating the presence of stem cells in the human OSE layer [104]. Later, OLCs were first obtained in vitro from human OSE culture [91]. These cells were cultured in the vicinity of fibroblasts and stem cells and therefore probably had the support of somatic cells. Fibroblasts express aromatase, which catalyzes the conversion of C (19) steroids to estrogen, and consequently can support the development of OLCs. Additionally, stem cells may have acted as granulosa cells. This study reported that some OLCs reached ~95 μm in diameter, and in some cases, formed a structure similar to zona pellucida, expressing oocyte-specific factors; however, they did not show early meiotic markers such as SCP3.

Differentiation of OSE cells into primary granulosa cells was reported prior to this study [105]. Subsequently, spontaneous differentiation of OSE cells from the ovaries of postmenopausal women was reported [92]. This study detected pluripotent very small embryonic-like cells (VSELCs) among OSE cells. Culturing these cells for three weeks led to an increase in their size and the formation of small oocyte-like structures, which is sometimes also showed a polar body. In some cases, a structure similar to a zona pellucida was observed. In other cases, large oocyte-like structures with a diameter of 130 μm were observed.

In a landmark study, White et al. (2012) optimized the method of isolating oogonial stem cells (OSCs) from the ovaries of humans and mice [67]. Isolation and maintenance of these cells that can produce functional oocytes, especially in humans, can be effective options in the treatment of infertility in women undergoing chemotherapy or suffering from POI. Transplantation of human OSCs (with the GFP marker) into the ovaries of adult mice produced GFP-positive oocytes (5–6 months after injection). Some of these oocytes developed into blastocysts with the GFP marker after IVF and embryo culture. This study was a significant advance in the detection of potential germ cells in mice and humans. However, problems persisted, especially concerning human oocyte-like structures. This is likely because these structures are always derived from cells that need somatic cells to develop and differentiate. The somatic cells provide the signaling as well as physiological needs of these structures to produce follicles, and eventually, functional oocytes with the ability to complete meiosis, fertilization, and embryonic development. Aggregation of these OLCs with somatic cells cultured from the human ovary can lead to the formation of oocyte-containing follicles with the capacity to grow and differentiate [106] into other groups that in turn isolate and differentiate into adult human ovarian OSCs [89]. An obvious difference noted between OLCs derived from oocyte differentiation of human VSELs was the lack of structures, similar to that of zona pellucida [90]. In a recent study, OLCs were collected from the VSELs of a patient with premature ovarian failure (POF) [93]. Interestingly, the proximity of the collected cells to sperm during IVF led to the spontaneous formation of structures similar to zona pellucida, expressing genes such as *OCT4* and *ZP3*. However, it was also noted that exposure to sperm did not lead to any fertilization in the structures.

### 3.3. Pluripotent Stem Cells

#### 3.3.1. Embryonic Stem Cells

##### Embryonic Stem Cells from Mouse

**Figure 4:**
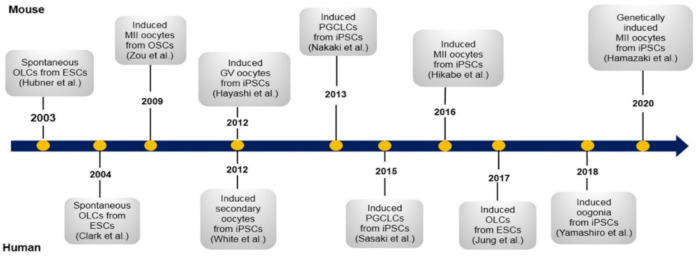
Landmark studies in mouse and human oocyte differentiation from pluripotent stem cells. Only a few of the most effective studies have been mentioned. These works, along with other efforts discussed in the text, provided a better understanding of how germ cells differentiate in humans and mice. OLCs: oocyte-like cells; ESCs: embryonic stem cells; OSCs: ovarian stem cells; GV: germinal vesicle; iPSCs: induced pluripotent stem cells; PGCLCs: primordial germ cell-like cells.

The first report on the generation of mouse oocytes from stem cells [76] dates back to the early 21st century (Figure 4). In this study, OLCs were collected from mouse embryonic stem cells (ESCs), however, fertilization and development of a biparental embryo and the birth of a healthy pup—which is the standard indicator for evaluating functional gametes—were not reported in this experiment. Years later, based on new observations and studies—which enabled in vitro maturation of PF [43] and fetal germ cells [107]—PGCs successfully matured into MII oocytes competent for fertilization and full-term development [45]. An integral characteristic of PGCs is the ability to retain totipotency. This characteristic is the result of highly coordinated gene functioning and epigenetic programming that must occur at both the DNA and histone levels [108]. In addition, during the induction of germ cells from embryonic stem cells, chromatin undergoes drastic changes in its architecture. These changes have been comprehensively studied before [109,110]. In order to create ideal conditions for obtaining PGCs in vitro, the developmental conditions of PGCs in vivo need to be successfully imitated. Numerous attempts have been made to mimic these conditions, including transplanting PGCs below the ovarian bursa [82] or into the kidney capsule [111].

**Figure 5:**
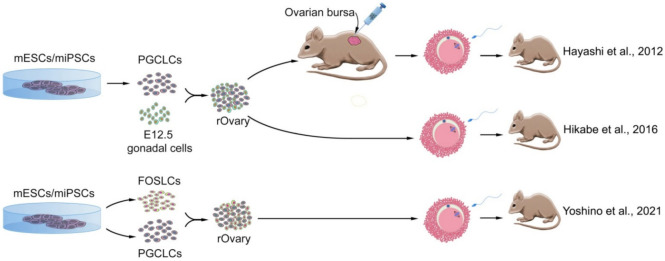
Successful methods of deriving oocytes in vitro from mouse pluripotent stem cells. Cells originated from mouse embryonic stem cells (mESCs) or mouse-induced pluripotent stem cells (miPSCs) developed into primordial germ cell-like cells (PGCLCs) and differentiated into oocytes either by aggregating with in vivo-derived fetal gonadal somatic cells or with fetal ovarian somatic cell-like cells (FOSLCs), after transplant into the ovarian bursa or by generating reconstituted ovary (rOvary) containing growing oocytes. All of these strategies led to producing live offspring [80,82,85].

Although functional oocytes were developed in the abovementioned studies, with both studies reporting the birth of healthy offspring from fertilization of these oocytes, nevertheless, most of the development of the oocyte progenitor cells—i.e., PGCs—took place in vivo (Figure 5). Morohaku et al. (2016) showed that more than 95% of secondary follicles grow abnormally in vitro, most likely in response to the marked difference in gene expression compared to in vivo conditions [45]. The authors speculated that some FBS ligands in the culture medium may have increased estrogen signaling levels, thereby affecting the expression of hundreds of genes. Adding estrogen receptor antagonists to the culture medium significantly improved the quality of secondary follicles and increased the number of follicles containing a primary oocyte.

Mouse ESCs were differentiated into ovary-like structures containing oocytes using a conditional medium prepared from the testes of neonatal mice [77]. This medium contained several types of growth factors, including Gdf-9, Bmp-4, SCF, LIF, and IGF-I, which resulted in the directed differentiation of ESCs into germ cells. These ovary-like structures were made of EBs derived from the differentiation of ESCs in the conditional environment. These factors are associated with germ cells in the testis, as well as with somatic cells such as Sertoli and Leydig. However, these factors are also associated with granulosa and theca cells in the ovary, additionally contributing to oogenesis regulation. In ovary-like structures, OLCs with one or two cell layers resembling normal granulosa cells were visible. This observation infers that the conditional medium containing the cultured testicular cells could also provide the necessary conditions for the formation and development of follicles. However, this study had its limitations, including the fact that the OLCs collected showed only a state similar to the early stages of oocyte growth.

A novel two-step system to study in vitro oogenesis was developed by Qing et al. (2007), in which ESCs differentiated into OLCs with the help of ovarian granulosa cells [78]. They showed that granulosa cells promote the induction of female PGCs through direct cell-to-cell contacts. However, this study was also not able to generate oocytes capable of completing meiosis and ultimately being fertilized. The authors correctly speculated that in current procedures, some factors necessary for oocyte differentiation must be lacking. As they predicted, and subsequent studies also confirmed, retinoic acid (RA), a metabolite of vitamin A that played an important role in inflammation, cell differentiation, and fetal development, and is produced by the kidneys and gonadal somatic cells, also acted as an integral external regulator for meiosis initiation. Studies in mice have shown that RA plays a key role in regulating the onset of meiosis [112]. Preliminary observations indicated that vitamin A deficiency in vitamin A-deficient mice led to a lack of differentiated germ cells, which was significantly compensated by vitamin A administration [113]. Moreover, when rat ovaries were cultured in a medium supplemented with RA, the number of meiotic cells significantly increased [114]. In this study, FLCs were obtained that contained GV oocytes, which, after maturing, were fertilized by sperm and successfully developed into morula and blastocysts.

##### Embryonic Stem Cells from Human

Advances from mouse research on germ cell induction from stem cells paved the way for similar achievements in humans. First, Clark et al. (2004) reported the differentiation of human ESCs (hESCs) into germ cells (GCs) in vitro [94]. These cells, like those in mice [76], spontaneously differentiated into EBs and expressed genes specific to mature GCs, such as *VASA*, *BOL*, *SCP1*, *SCP3*, *GDF9*, and *TEKT1* (Table 1). Although the differentiation of hESCs into GC precursors occurred spontaneously, it was soon found that the addition of recombinant BMP4 to the culture medium could stimulate the differentiation of hESCs and the formation of EBs expressing VASA and SYCP3 in a directed manner. Further, the addition of BMP7 and BMP8b could increase the effects of BMP4 [115]. It was found that mouse embryonic fibroblast (MEF) feeders and basic fibroblast growth factor (bFGF) led hESCs to differentiate into GCs at a high rate, resulting in the expression of DDX4 and OCT3/4 genes in ~70% of the cells. And notably, as differentiation progressed, more than 90% of the cells expressed meiotic genes—such as *SYCP3* and *MLH1*—ultimately differentiating a large population of germ-like cells from hESCs [116]. In another study, hESCs formed oocyte-like structures through RA treatment [95]. However, antibody testing against the zona pellucida proteins did not show the presence of this structure around the oocytes.

#### 3.3.2. Induced Pluripotent Stem Cells

##### Induced Pluripotent Stem Cells from Mouse

Female ESCs or iPSCs were differentiated into functional oocytes under special conditions [82]. ESC- or iPSC-derived primordial germ cell-like cells (PGCLCs) were co-cultured with gonadal somatic cells and formed ovary-like structures. These reconstructed ovaries (rOvaries) were then transplanted into the ovarian bursa of an infertile mouse. With this strategy, fully-grown GV oocytes were produced, developed into mature oocytes, fertilized by IVF, and subsequently birthed healthy offspring after embryo transfer. Until then, the production of artificial oocytes from PGCs, which could fertilize and produce healthy pups, was only possible by transplanting PGCs into other mice [111,117]. It appears that grafting to the ovarian bursa is essential for obtaining functional oocytes. This is because, in studies that did not do such a transplant, the resulting embryos were not able to develop to term, probably in response to the formation of abnormal follicles and insufficient growth of oocytes in vitro [118].

Hikabe et al. (2016) differentiated ESCs/iPSCs first into EpiLCs, then aggregating them with 12.5-day-old embryonic gonadal somatic cells to form rOvaries (Figure 5) [85]. The resulting oocytes reached the 2-cell stage embryos by IVF, with 3.5% of them successfully developing to offspring after transfer to foster mothers. Inducing in vitro differentiation without the involvement of somatic cells can be useful for better understanding the mechanisms that control the differentiation of female GCs. Identifying the genes—such as *Dazl*—that control the development and differentiation of GCs and how they are expressed made creating fully defined environments possible. According to Yu et al. (2009), overexpression of this gene in mESCs induces their differentiation into GCs [79], and produces both motile spermatozoa and oocytes in vitro, with no need to form EBs, as previous studies considered necessary [76,94,119,120]. By comparing gene expression in mESCs and GCs, it was found that some genes are expressed in mESCs but are silenced during the differentiation process of GCs. One of these genes was *Nanog*, which is essential in the self-renewal of mESCs. However, its expression decreases during GC differentiation and is completely absent in GCs that strongly express *Dazl*. In addition, the reduction of Dazl expression with siRNA showed that the expression of this gene was necessary to regulate the expression of other important genes such as Stella, Mvh, and OSE.

Nakaki et al. (2013) showed that the overexpression of *Prdm1* (also known as *Blimp1*), Prdm14, and Tfap2c induced the differentiation of EpiLCs, derived from mESCs or iPSCs, into PGCLCs [83]. Even overexpression of Prdm14 alone was sufficient to differentiate EpiLCs into PGCs. Induction of differentiation into PGCs occurred through overexpression of these transcription factors independent of Bmp4. This was because the chemical inhibition of cytokine signaling by LDN193181 did not inhibit overexpression of the three genes (i.e., *Prdm1*, *Prdm14*, and *Tfap2c*). In addition, overexpression of transcription factors increased the expression of key genes for the differentiation/development of PGCs, including *Blimp1*, *Prdm14*, *Tfap2c*, *Nanos3*, *Stella*, *Pou5f1*, *Sox2*, and *Nanog*, and decreased the expression of epigenetic modifiers such as Dnmt3a and Dnmt3b. At the same time, another study confirmed the key role of these three factors in the differentiation of PGCs [121]. This study also showed the simultaneous expression of three factors Blimp1, Ap2ɣ (expressed by the *Tcfap2c* gene), and Prdm14, when conducted as indirect induction of PGCLCs in vitro, completely replaces cytokines. A closer look at the genetic network provided a more accurate picture of how a unique epigenetic program is regulated in GCs.

Subsequent research revealed that TFs such as Nanog alone could induce the expression of key genes for PGC differentiation in such a way that EpiLCs could directly differentiate into PGCLCs without Bmp4 mediation [84]. Nanog binds to and activates *Prdm1* and *Prdm14* enhancers in EpiLCs and in vitro. Blimp1, encoded by the *Prdm1* gene, then directly induces *Tfap2c*. This study revealed that the overexpression of Nanog, rather than *Sox2* or *Oct3/4*, alone and independently of the cytokine signaling pathway, induced differentiation of PGCLCs from EpiLCs. In vivo, removal of Nanog also prevents the induction of PGCLCs by Bmp4. Therefore, Nanog cooperation is essential for inducing germ cell fate in vivo. This research showed that it is indeed possible to create female GCs through only induction with key TFs and without the need for cytokine signaling pathways.

PGCLCs, in addition to maintaining the ability to differentiate, passively and gradually erase DNA methylations at the genomic level and obtain a level of 5 mC that is similar to GCs in gonads [109]. Thus, PGCLCs have the inherent capability for epigenetic reprogramming; this ability being independent of embryonic gonads. Subsequently, research by Miyauchi et al. (2017) showed that Bmps and RA work together to direct PGCs or PGCLCs derived from ESCs or iPSCs toward fetal primary oocytes. Further, this can be done without the need for induction from gonadal somatic cells [86]. This in vitro system represents the key processes of germ cell development, including PGC specification, proliferation, epigenetic reprogramming, and female germ cell determination under defined culture conditions, and can provide a good platform to study the underlying mechanisms of such processes.

By comparing EBs from iPSCs derived from granulosa cells, fibroblasts, and G4 ESCs, Anchan et al. (2015) found that EBs obtained from granulosa cell-induced iPSCs (GriPSC-EB) synthesized ten times more estradiol (E2) than other cell lines [122]. In contrast, ESC-EBs mostly synthesized progesterone (P4), while iPSC-EBs synthesized none of them. Moreover, GriPSC-EBs expressed ovarian markers such as Amhr, Fshr, Cyp19a1, and ER, as well as gametogenesis markers such as Mvh, Dazl, Gdf9, Boule, and Zp1, more than other EBs in the same culture medium. In a landmark study, Tian et al. (2019) were able to induce somatic granulosa cells isolated from the ovaries of adult mice into PSC, and then differentiate them into PGCLCs and further to functional oocytes [87]. This differentiation was made entirely by chemicals such as Rock inhibitor and crotonic acid.

In a major development, Hamazaki et al. (2020) then identified a set of transcription factors that included the basic gene regulatory network in the development of PGCs in mice. By manipulating the genes encoding these factors, they differentiated PSCs—directly and without converting to PGCs—into OLCs capable of fertilization and early embryo formation [88]. To determine the gene regulatory network, these researchers first differentiated ESCs into PGCLCs using the system they described earlier [85]. Targeting eight genes, *Figla*, *Sohlh1*, *Lhx8*, *Nobox*, *Stat3*, *Tbpl2*, *Dynll1*, and *Sub1*, in mESCs, stopped their differentiation before or around primordial-to-primary-follicle transition (PPT). Among these eight genes, the presence of four genes, *Nobox*, *Figla*, *Tbpl2*, and *Lhx8*, is essential for the direct induction of oocyte-like cell (DIOL), and no DIOL from the ESCs was observed without either of these genes. Induction of mouse embryonic fibroblasts (MEFs) with these eight genes did not result in the formation of any DIOL. However, induction of iPSCs, induced from adult mouse tail fibroblasts, yielded the same results as ESC induction, which meant that having a pluripotent status may play a key role in inducing DIOLs. This study also showed that genes essential for the differentiation of PGCs, such as *Prdm1*, *Prdm14*, and *Tfapt2c*, were not expressed in the DIOL induction process. Further, even ESCs lacking the *Prdm1* gene, which could not differentiate into PGCs, can be successfully differentiated into DIOLs. Therefore, the differentiation of DIOLs from ESCs did not require a transition to a PGC intermediate state. After 11 days of culture, DIOLs evolved into GV oocytes and were able to develop into MII oocytes (MII-DIOL) under IVM conditions. Finally, this study showed that MII-DIOLs have the potential to fertilize and form early embryos.

However, MII-DIOLs showed serious chromosomal abnormalities during the developmental process. After GVBD, the chromosomes separated abnormally, indicating a lack of meiotic recombination. In addition, the genes necessary to enter the meiotic prophase were not upregulated. The polar body was also absent in most MII-DIOLs. Finally, the developmental potential of embryos obtained from DIOLs was very limited, with less than 4% of them reaching the 4/8-cell stage. This was attributed to aneuploidy. However, the results of these experiments showed that oocytes could be derived directly from PSCs if the gene regulatory network was reconstructed with specific transcription factors.

In the most recent study conducted in this field, Yoshino et al. (2021) introduced a system in which the ovarian somatic cell environment is reconstructed using mouse PSCs [80]. Through a series of cultures conditioned to stimulate differentiation into gonadal somatic cells, mESCs became fetal ovarian somatic cell-like cells (FOSLC), which express Nr5a1, a specific marker for differentiation into gonadal somatic cells. Transplantation of mESC-derived PGCLCs into the vicinity of these cells caused their aggregation and entry of PGCLCs into meiosis and, consequently, the growth of oocytes and the development of follicles derived from FOSLCs in the culture medium. Oocytes obtained from this system were capable of fertilization and development and generated healthy and fertile offspring (Figure 5). Thus, functional follicular structures capable of producing functional oocytes can be reconstructed in vitro without the need for embryonic gonads.

##### Induced Pluripotent Stem Cells from Human

After succeeding in creating iPS cells in humans [123], researchers were then able to differentiate hiPSCs and hESCs into female PGCs, with the support of human fetal gonad stromal cells [124]. This particular study reported that differentiation in culture medium for seven days significantly increased the expression of germ cell-specific factors, PRDM1, DPPA3, and DAZL, in induced PGCs. In these experiments, however, GCs remained in the early stages of differentiation and did not develop into meiotic and fertilizing oocytes. Kee et al. (2009) modulated the differentiation and development of human GCs by manipulating the expression of GC-specific genes encoding cytoplasmic RNA-binding [125]. The researchers discovered that overexpression of genes such as *DAZL* and *BOULE* triggered the formation of PGCs. Moreover, overexpression of *DAZ* family genes pushed PGCs forward to advanced stages of both meiosis and haploid cell formation. This study significantly improved the differentiation of PGCs induced from hESCs into post-meiotic stages, which barely happened in previous studies. Overexpression of *DAZL* and *BOULE* [126], and *VASA* and *DAZL* [127] in hPSCs had similar results and led to the formation of post-meiotic PGCs and GCs. In addition, overexpression of STELLA, accompanied by induction with RA, increased the expression of genes involved in GC development and differentiation from hESCs [128]. However, in none of these studies were functional oocytes obtained. Even the early stages of oogenesis did not occur in these experiments.

The naive human stem cell medium (NHSM) facilitated the transition of primed hPSCs to the naive state in a transgene-independent manner [129]. Later, hPSCs in a four-inhibitor-containing medium (4i)—which was adopted and modified from NHSM conditions—were transited to the naive state and then differentiated into hPGCLCs by induction of BMP2/4 [97]. This study also showed a fundamental difference between humans and mice in the regulation of PGC differentiation, so that BLIMP1—which is an essential transcription factor for germ cell lineage specification in mice—is downstream of SOX17 in humans, and represses endodermal and mesodermal genes during hPGCLC specification.

Induction of hiPSCs by treatment with ActA and GSK3β inhibitors differentiated them into incipient mesoderm-like cells (iMeLCs) [98]. Mesoderm-like cells, and then PGCLCs, were obtained by treating hESCs or hiPSCs with ActA, bFGF, and BMP4, respectively [98]. Collectively, these studies explained the conditions for the isolation, culture, and differentiation of human PGCLCs, and paved the way for further studies and the discovery of mechanisms that control the GC fate in humans.

**Figure 6:**
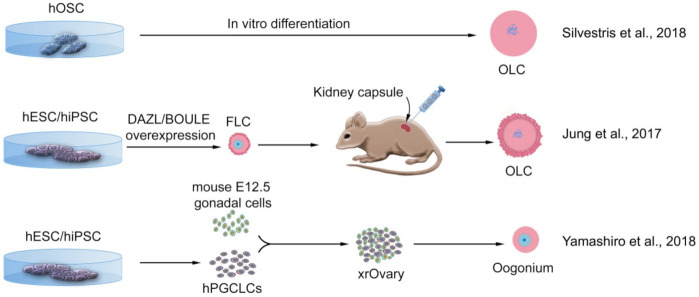
Successful methods of deriving oocyte-like cells in vitro from human pluripotent stem cells or ovarian stem cells. Cells that originated from human oogonial stem cells (hOSCs) directly differentiated into oocyte-like cells (OLCs). Human embryonic stem cells (hESCs), or human-induced pluripotent stem cells (hiPSCs), developed into follicle-like cells (FLCs), transplanted into the mouse kidney capsule, and developed to OLCs; or, hESCs or hiPSCs differentiated into primordial germ cell-like cells (PGCLCs), aggregated with mouse fetal gonadal somatic cells, form xenogenic reconstituted ovary (xrOvary) and further differentiated into pre-meiotic oogonium. None of these strategies led to producing meiotic oocytes [90,96,100].

Serious limitations in these types of studies—when compared to mice—are the technical and ethical impediments of using human tissues. The major success in mice was the acquisition of OLCs using co-culture with 12.5-day-old embryonic gonadal somatic cells. Therefore, it has been desirable to achieve an approach in which complete differentiation occurs in vitro and does not require any human tissue. For example, by manipulating the genes necessary for germ cell development and differentiation, Jung et al. (2017) were able to exit hESCs from pluripotency and direct their differentiation into GCs and meiosis [96]. Overexpression of *BOULE* in hESCs increased the expression of CDC25A, an important regulator of meiosis I progression in oocytes. By over-expressing *DAZL* and *BOULE* simultaneously, more hESCs were placed on the 4n-chromosomal state, indicating entry into meiosis (Figure 6).

Further to the above, by adding GDF-9 and BMP15 to the culture medium, follicle-like cells (FLCs) emerged in which zona pellucida were visible around OLCs. Transplantation of these follicle-like structures into the mouse kidney capsules for 46 days showed that these cells expressed genes such as *NOBOX* and *AMH* in their proper place, the oocyte nucleus, and the surrounding granulosa cells, respectively. This finding indicated that these cells retained their identity as ovarian follicles even in the kidney capsule. However, in this study, mature oocytes with the fertilization capacity were not obtained. This was probably because the presence of human somatic gonadal cells is necessary for the development of oocytes and their transition to the second meiosis. Yamashiro et al. (2018) combined these cells with mouse embryonic ovarian somatic cells to produce xenogeneic reconstituted ovaries (xrOvaries) [100]. First, hiPSCs with male karyotype were differentiated into iMeLCs and then into hPGCLCs. These cells were then co-cultured with mouse embryonic somatic cells for 10 weeks, resulting in hPGCLCs undergoing epigenetic reprogramming to differentiate oogonia/gonocyte-like cells (Figure 6). Expression of key genes for meiotic prophase entry began after four months of culture. The cells then being differentiated into cells similar to RA-responsive fetal GCs, the immediate precursor of fetal oocytes. Therefore, hPSCs in the primed state could differentiate into GCs in response to a reliable protocol being available to achieve these cells [130]. However, in this study again, a fertilizable MII oocyte capable to form an embryo was not achieved, and this was probably due to the incompatibility of mouse somatic cells with human iPSCs. Thus, the question of the limitation of obtaining somatic support cells from human organs for technical and ethical reasons remains. A possible solution to this limitation would be to differentiate iPSCs into granulosa cells [131].

## 4. Haploidization in Somatic Chromosomes

Somatic cell haploidization is the process by which meiosis is induced into the 2n chromosomal nucleus of a somatic cell, and the number of chromosomes is reduced to n [132]. Significant successes in the transfer of somatic cell nuclei into oocytes and their full development to maturity and fertility in animals have shown that somatic nuclei in oocytes are extensively remodeled. One such observation was noted in experiments that showed that round spermatids injected microsurgically into adult mouse oocytes fully supported development [133,134]. Even the injection of secondary spermatids resulted in the complete development of the mouse embryo [134]. These experiments used cells that had previously gone through a stage of meiosis. Although these results assisted in understanding the remodeling mechanism required for transplanting nuclei by oocytes, differences in how oocytes interact with these nuclei and somatic nuclei should not be overlooked. However, subsequent experiments showed that even the nucleus of mitotic cells could be reprogrammed in mouse oocytes and support full development [135,136].

The MII oocyte cytoplasm can remodel the nucleus of somatic cells in such a way that it can pass through it without stopping at the cell cycle checkpoint—which normally prevents the somatic nucleus from entering metaphase before completing the DNA synthesis phase [137]. In 2001, this feature of the MII oocyte was used to reconstruct a woman’s oocytes that did not survive ovarian stimulation, by transferring the nucleus of the individual’s cumulus cells to the donor’s enucleated oocytes [138].

The somatic cell haploidization has been performed in two other ways. In one method, the somatic cell in the G0/G1 phase merges with a GV oocyte and in the G2/M phase, where the number of somatic cell chromosomes decreases by half as the oocyte matures and a polar body is extruded [139]. In another method, the somatic cell is at G2/M and enters the GV oocyte which is in the G2/M phase [140,141,142]. Here, two stages of reduction are needed. In the first, the number of somatic cell chromosomes is reduced to n, and in the second, the number of oocyte chromosomes is halved, and a polar body is extruded at each stage. The second method was used to haploid human cumulus cells using GV oocytes [141]. In addition, MII oocytes were used in humans to haploid cumulus cells [143]. None of these experiments reported normal fetal development and birth, mainly due to severe abnormalities in chromosome segregation and aneuploidy, and thus, such strategies for treating infertility in humans were completely abandoned [138,144].

**Figure 7:**
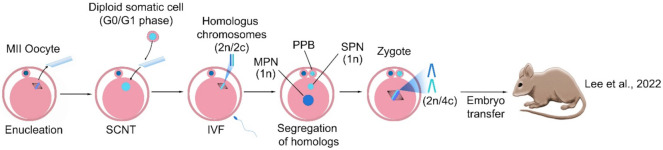
Induction of haploidy in a somatic cell by the mature oocyte. The G0/G1 somatic cell was transferred into the enucleated MII oocyte and the de novo spindle consisting of somatic homologous chromosomes was reconstructed. Fertilization triggered both homologous segregation and extrusion of pseudo polar body (PPB), and further, generation of the somatic pronucleus (SPN) and male pronucleus (MPN). The haploid somatic and sperm pronuclei were formed in the reconstructed zygote. The embryo with the somatic haploid can then develop and produce live offspring. SCNT: somatic cell nuclear transfer [145].

Recently, the present study revisited the haploidization of diploid somatic chromosomes using the somatic cell nuclear transfer (SCNT) [145]. Through it, it was confirmed that meiotic spindles were formed after the transfer of G0/G1 somatic cells into enucleated MII oocytes and succeeded to generate the somatic haploid chromosome (Figure 7). What happens here is that if the somatic cell’s nucleus in the G0/G1 phase of the cell cycle is transferred into an enucleated MII oocyte, because DNA synthesis does not occur in this nucleus, chiasmata do not form and the somatic cell chromatin forms metaphase chromosomes. Then, after the sperm enters and the oocyte is activated, a set of somatic chromosomes emerge from the cytoplasm in the form of a pseudo-polar body, thus haploidizing the somatic cell nucleus. The remaining set in the cytoplasm and the set of sperm chromosomes form the female and male pronuclei, respectively, and the zygote is formed. An average of 76% of the homologous chromosome was properly segregated in somatic haploid embryos, which were established embryonic stem cells (ESCs) and produced offspring.

This study suggested that the non-random chromosome segregation of the somatic nucleus could occur in MII oocytes, further confirming that the present study using the modified SCNT protocol with various combinations of chemicals or proteins, could assist with proper chromosome segregation. Further, the results demonstrated that the contribution of somatic chromosomes in somatic haploid embryos was either maternal or paternal alleles of the somatic genome. The somatic haploid embryos produced live offspring in this study, but the efficiency was very low. This is explained by chromosome loss in somatic haploid embryos because of failure of somatic chromosome segregation, the limitations of the reprogramming and development in SCNT—such as inappropriate nuclear reprogramming and abnormal placental development—and the mismatch of the reprogramming cycle between sperm and somatic nucleus potentially block the normal embryo development and offspring production. Even with the limitation of low efficiency, it is the first study to generate the birth from the somatic haploid embryos, claiming that their finding could be a new strategy to generate oocytes carrying somatic genomes.

## 5. Challenges and Future Scenarios

There have always been challenges in inducing the differentiation of somatic cells into functional oocytes. One of these challenges has been the low rate of differentiation of PGCLCs. For example, the rate of differentiation of PGCLCs from iPSCs in mice was not more than 15% [81]. In addition, the epigenetic abnormalities observed in the differentiation of iPSCs call into question the efficiency of using these cells to produce oocytes for human use. The complete dependence of PGCLCs on granulosa cells to develop and become oocytes has been associated with ethical and technical problems and has posed a serious impediment to the use of this method in the treatment of infertility. Abnormal meiotic divisions leading to aneuploidy have also marginalized the use of somatic cell haploidization techniques. Ovarian stem cells (OSCs) and VSELCs survive in the ovaries of women undergoing cancer therapy and can differentiate into oocytes, enabling fertility for these women. But these cells are difficult to isolate and only very few are present in the ovarian tissue. In addition, they can only be used specifically for the patient. Therefore, despite their benefits, they are not an ideal option for the global treatment of infertility. Further research is needed to properly understand and address these issues.

The hESCs became virtually an endless source for oocyte production in the laboratory. The use of iPSCs, although controversial, also reduced the ethical implications of using hESCs. However, it should not be overlooked that the use of these cells is not without risks. For example, undesirable epigenetic and genetic changes have been reported in both hESCs and hiPSCs [146,147], some of which can even alter the differentiation propensity of these cells [148]. There are limitations to using these cells even when they are free of undesirable genomic mutations. hESCs cannot be used patient-specifically to produce oocytes. Instead, using iPSCs can alleviate this problem to some extent. iPSCs, however, usually carry some of the epigenetic memory of the somatic tissue from which they are derived, and this can affect their ability to differentiate [149]. The iPSCs, alternately, sometimes contain mitochondrial mutations that can be transmitted to the resulting oocytes and embryos [150].

The use of iPSCs as the primary source of oocyte induction is preferable to the use of ESCs, especially in humans. This is because access to iPSCs is technically easier and less risky. In addition, iPSCs can be patient-specific, with the cells derived from them being more compatible with the individual’s immune system. However, as mentioned, the rate of induction of differentiation of these cells into oocytes is very low.

In the future, this problem can be solved by choosing a cell type closer to oocytes. The differentiation of human PSCs has only been achieved up to the oogonium. Thus, the formation of artificial oocytes in humans must be performed either by haploidization of the adult somatic cells, or by transferring the oogonium nucleus obtained from PSCs to the oocytes; of course, the latter is preferable because it is cheaper, faster, and easier for the patient [151]. For example, as research by Tian et al. (2019) showed, mouse granulosa cells can be converted to PSCs by chemical induction, and these cells differentiate more favorably into functional oocytes [87]. In infertility clinics, the granulosa cells that accompany oocytes are usually discarded after IVF. The use of these cells and their chemical differentiation into iPSCs and then oocytes could potentially be considered an effective treatment option as long as they are genetically and epigenetically safe before differentiation into oocytes. iPSCs derived from a patient’s somatic cells can carry genetic mutations or epigenetic abnormalities, and therefore pose a risk of transmitting some diseases to the next generations. Genetic disorders such as aneuploidies and CNVs can enter the genome of iPSCs during reprogramming, differentiation, and culture [152]. Therefore, screening these disorders before clinical use of iPSCs is essential [153].

**Figure 8:**
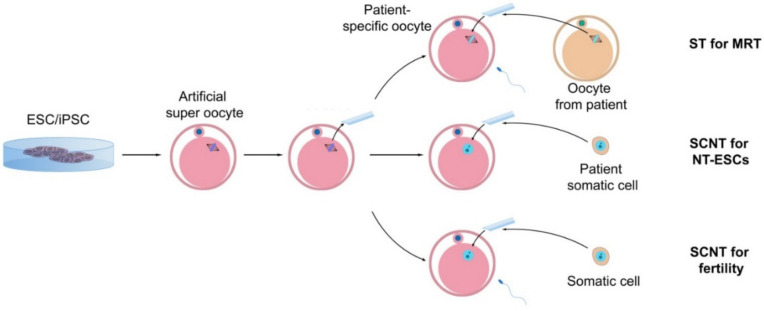
Application of artificial super-oocytes for patient-specific stem cells and infertility treatment. Oocytes can be obtained from pluripotent stem cells, and after enucleating, can be used either for spindle transfer (ST) for mitochondrial replacement therapy (MRT), transfer of the somatic cell nucleus (SCNT) from the patient’s cells for embryonic stem cell (ESC) culture, or nuclear transfer-derived ESCs (NT-ESCs) for fertility treatment and generation of the new oocytes.

Correction of some of these anomalies is now more possible with the availability of technologies such as CRISPR. In addition, genome editing is much easier in iPSCs than in zygotes. Rapid advances in genome editing have raised hopes that many human genetic abnormalities can be addressed before pregnancy. The use of this system in gene therapy is rapidly in progress [154]. A wide variety of Cas enzymes has been developed that can greatly increase the efficiency of the genome editing system [155]. Adding to this, numerous new technologies and software have been developed to increase the accuracy of sgRNA design [156]. In addition to being effective in generating precise mutations and in vitro models for diseases, CRISPR can also be extremely effective in correcting genetic mutations in patient-specific iPSC models [157]. For example, Wang et al. (2020) used the CRISPR/Cas9 system to correct a mutation in the CSB/ERCC6 gene that causes Cockayne syndrome in iPSCs derived from a patient’s fibroblasts [158]. Moreover, CRISPR can edit multiple mutations simultaneously in human cells [159]. Attempts have been made to induce clinically “safer” iPSCs [160]. These iPSCs, in which genetic and epigenetic defects are minimized, can be used as universal donor cells and differentiate into oocytes being used for multiple purposes (Figure 8). These “artificial super oocytes” can provide an endless and safe source for restoring the hope of healthy fertility to women who have been deprived of the gift of conception.

## 6. Conclusions

The summarized results have raised great hopes for the treatment of infertility in women who are unable to produce functional oocytes. In the laboratory thus far, human PSCs have been differentiated into pre-meiotic oocytes (oogonia) only. However, these oocytes had no potential for fertilization and embryonic development, and therefore, no report of functional oocyte production in humans has been published. However, given the remarkable success in laboratory animals, it is likely that we shall soon witness significant advances in the use of stem cells in the treatment of infertility in humans.

Basic research in stem cell biology has provided an improved understanding of the cellular and molecular mechanisms that regulate the differentiation of the cells in vitro. Because such research has aided efficient methods and protocols being developed for inducing PSC differentiation into GCs. These methods, based on the recognition of molecular markers specific to each stage of germ cell differentiation, have made it possible to separate these cells from other cells. A better understanding of the physiological and genetic characteristics of these cells also paved the way for induction of their differentiation through the use of small molecules modulating the action of transcription factors or manipulating the expression of genes in vitro.

Deficiencies in infertile women’s oocytes can be due to either nuclear or cytoplasmic defects. Research shows that there is a clear difference between the quantity, distribution, and morphology of cytoplasmic organelles between poor-quality and good-quality oocytes [161]. Methods have been used to correct this defect in oocytes, including nuclear transfer, spindle transfer, polar body transfer, and pronuclear transfer [162,163].

Among the abovementioned methods, spindle transfer has led to the birth of a human baby. What makes this method so important is that it can be used to prevent the inheritance of pathogenic mutations in human mtDNA. In this method, first performed in non-human primates, the nuclear genome, which here is in the form of metaphase II (MII) chromosomes, is removed from an oocyte with mutant mtDNA and transferred to an oocyte with WT mtDNA whose nuclear genome has already been removed (cytoplast; Figure 8). The reconstructed oocyte is then fertilized with the partner’s sperm and continues to develop after being transferred to the mother [164]. This method was then applied to human MII oocytes [165,166,167] and used to prevent the transmission of mitochondrial genetic disease (Leigh syndrome) to the human embryo [168]. The first report of a human baby born using this method was published in 2017 [169]. However, what limits this method is that healthy cytoplasts with WT mitochondria must always be obtained from oocyte donation.

In oocyte donation, invasive methods are usually used and there will always be risks for the donor. Therefore, it seems necessary to use an alternative method without invasion and ethical concerns. With the development of methods for creating oocytes from the sources available in laboratories, it will always be possible to have enough cytoplasts.

## Figures and Tables

**Table 1:** In vitro oogenesis and its markers from different sources in mice and humans.

Mouse			
Source	Outcome	Markers for Selection	References
OSC	MII oocyte	*Scp3, Spo11, Dmc1*	[68]
	MII oocyte	*Oct4, Blimp1, Dazl, Stella, Mvh, Fragilis, Rex1*	[44]
	FGSCs	*Ddx4, Dazl, Blimp1, Stella, Fragilis*	[72]
	Oocyte	*Oct4, Ddx4, Stella, Nobox, Sohlh1, Zp3*	[73]
VSELC	OLC	*Mvh, Stra8, Msy-2*	[74]
	OLC	*Ddx4, Dazl, and Zp3*	[75]
mESC	OLC	*Zp2, Zp3, Figα*	[76]
	OLC	*Zp3, Scp3, Figα*	[77]
	OLC	*Zp3, Gdf9, Figα*	[78]
	OLC	*Zp1, Zp2, Zp3, Gdf9*	[79]
	MII oocyte	*Prdm1, Stella, Ddx4, Scp3*	[80]
miPSC	PGCLC	*Prdm1, Stella*	[81]
	GV oocyte	*Prdm1, Prdm14*	[82]
	PGCLC	*Prdm1, Prdm14, Tfap2c, Nanos3, Stella*	[83]
	PGCLC	*Prdm1, Nanos3*	[84]
	MII oocyte	*Stella, Dazl*	[85]
	Oocyte	*Scp3, Stra8, Nobox*	[86]
	MII oocyte	*Prdm1, Dazl, Vasa*	[87]
	MII oocyte	*Gdf9*	[88]
**Human**			
OSE	OLC	*ZP, CK18*	[51]
	OLC	*SSEA-4*	[89]
OSC	OLC	*GDF-9, SYCP3*	[90]
	Oocyte	*DDX4, KIT, YBX2, NOBOX, LHX8, GDF9, ZP1 ZP2, ZP3*	[67]
VSELC	OLC	*OCT4A/B, VASA, ZP*	[91]
	OLC	*c-KIT, DAZL, GDF-9, VASA, ZP4*	[92]
	OLC	*OCT4A, ZP3*	[93]
hESC	OLC	*VASA, BOL, SCP1, SCP3, GDF9, TEKT1*	[94]
	OLC	*VASA, SSEA-1, DAZL*	[95]
	Primary oocyte	*OCT4, NANOG, PRDM14, VASA*	[96]
hiPSC	PGCLC	*PRDM1, STELLA*	[97]
	PGCLC	*PRDM1, PRDM14, TFAP2C, T, SOX17, SOX15*	[98]
	PGCLC	*PRDM1, STELLA, KLF2, TFAP2C*	[99]
	Oogonium	*DAZL, DDX4, REC8, SYCP3, STRA8*	[100]

OSC: ovarian stem cell; VSELC: very small embryonic-like cell; FGSC: fetal gonadal stem cell; OLC: oocyte-like cell; PGCLC: PGC-like cell; OSE: ovarian surface epithelium.

## Data Availability

Not applicable.
